# Nano-Formulation Endows Quorum Quenching Enzyme-Antibiotic Hybrids with Improved Antibacterial and Antibiofilm Activities against *Pseudomonas aeruginosa*

**DOI:** 10.3390/ijms23147632

**Published:** 2022-07-11

**Authors:** Kristina Ivanova, Aleksandra Ivanova, Javier Hoyo, Silvia Pérez-Rafael, Tzanko Tzanov

**Affiliations:** Grup de Biotecnologia Molecular i Industrial, Department of Chemical Engineering, Universitat Politècnica de Catalunya, Rambla Sant Nebridi 22, 08222 Terrassa, Spain; kristina.ivanova@upc.edu (K.I.); aleksandra.asenova@upc.edu (A.I.); javier.hoyo@upc.edu (J.H.); silvia.perez.rafael@upc.edu (S.P.-R.)

**Keywords:** gentamicin, acylase, nano-spheres, quorum sensing inhibition, antibacterial and antibiofilm activity

## Abstract

The emergence of antibiotic resistant bacteria coupled with the shortage of efficient antibacterials is one of the most serious unresolved problems for modern medicine. In this study, the nano-hybridization of the clinically relevant antibiotic, gentamicin, with the bacterial pro-pathological cell-to-cell communication-quenching enzyme, acylase, is innovatively employed to increase its antimicrobial efficiency against *Pseudomonas aeruginosa* planktonic cells and biofilms. The sonochemically generated hybrid gentamicin/acylase nano-spheres (GeN_AC NSs) showed a 16-fold improved bactericidal activity when compared with the antibiotic in bulk form, due to the enhanced physical interaction and disruption of the *P. aeruginosa* cell membrane. The nano-hybrids attenuated 97 ± 1.8% of the quorum sensing-regulated virulence factors’ production and inhibited the bacterium biofilm formation in an eight-fold lower concentration than the stand-alone gentamicin NSs. The *P. aeruginosa* sensitivity to GeN_AC NSs was also confirmed in a real time assay monitoring the bacterial cells elimination, using a quartz crystal microbalance with dissipation. In protein-enriched conditions mimicking the in vivo application, these hybrid nano-antibacterials maintained their antibacterial and antibiofilm effectiveness at concentrations innocuous to human cells. Therefore, the novel GeN_AC NSs with complementary modes of action show potential for the treatment of *P. aeruginosa* biofilm infections at a reduced antibiotic dosage.

## 1. Introduction

The emergence of multidrug-resistant bacteria in hospital and community settings has jeopardized the efficacy of the accessible antibiotic treatments, resulting in high mortality and morbidity rates, increased treatment costs and low confidence in healthcare services and providers [1]. Antimicrobial resistance (AMR) is one of the greatest healthcare threats, projected to cause 10 million deaths annually in 2050, unless action is not taken [2]. According to the European Centre for Disease Prevention and Control, the AMR consequences account for €1.5 billion annual health-care expenditure in Europe and, if not contained, the economic cost in terms of global production loss is estimated to reach $100 trillion [2,3].

*Pseudomonas aeruginosa* (*P. aeruginosa*) is one of the most concerning pathogens, included in the WHO’s threat list, which is associated with respiratory tract, blood stream, wound, skin and urinary tract infections [4]. Cationic antibiotics, including gentamicin, tobramycin and colistin, are administrated as the last resort treatments for patients with *P. aeruginosa* infections. However, this bacterium grows predominantly in a biofilm phenotype, where the killing properties of these conventional drugs are restricted [5,6,7]. Biofilms are surface-attached communities of bacterial cells surrounded by a self-produced extracellular polymeric matrix that exhibits up to 1000 times greater antibiotic tolerance, compared to the free-floating cells [7]. The biofilm formation is under the control of a cell-to-cell communication process known as quorum sensing (QS). The QS coordinates *P. aeruginosa* pathogenicity in a cell density-dependent manner, being the concentration of small signaling molecules, called acyl homoserine lactones (AHLs), in the extracellular environment of key importance for the biofilm establishment [8]. The interruption of the QS by quorum quenching enzymes (QQE), degrading the AHLs, or chemical QS inhibitors (QSIs) inactivating specific QS receptors of the *Pseudomonas* strains, has been employed to suppress bacterial virulence [9], and to boost the efficacy of the existing antimicrobials [10,11,12]. Recently, the nano-sized transformation of anti-QSIs have gained significant attention for improving their stability, therapeutic potential and biocompatibility [13]. Most of the developed nano-formulations, however, are based on QSIs that possess certain limitations, such as low stability, toxicity and high probability to evoke resistance [14]. From other sites, the use of QQE with enhanced efficacy and selectivity against the targeted bacteria, as well as a lower risk of resistance emergence, is scarcely reported [15,16].

Herein, we report for the first time on the development of novel and highly efficient nano-hybrids of the anti-QS enzyme acylase and gentamicin with enhanced bactericidal and antibiofilm efficacies against Gram-negative *P. aeruginosa*. A one-pot, fast, water-based, sonochemical synthesis approach, performed under mild conditions and without the need for any chemical modifications of the starting materials, was employed for the nano-hybridization of the actives. The nano-formulation of gentamicin is envisaged to endow it with penetrability into the *P. aeruginosa* biofilm structure and with membrane disruption capacity, leading to complete bacterial eradication at low selective pressure for the emergence of resistance [16,17,18,19]. Furthermore, the antibiotic combination with acylase, specifically targeting the pro-pathological cell-to-cell communication of *P. aeruginosa*, is a new “adjuvant” strategy for making bacteria less virulent and more susceptible to low concentrations of the nano-formulated drug. Contrary to the common practices for drugs’ discovery, the single-step nano-hybridization of a clinically relevant antibiotic with QQE to boost its efficiency will save on the time- and resource-consuming screening for new antimicrobials.

The hybrid acylase/gentamicin nano-spheres (GeN_AC NSs) developed in this work will be characterized in terms of their ability to attenuate the QS-regulated virulence and impede the drug-resistant *P. aeruginosa* biofilm formation. The bactericidal activity of the GeN_AC NSs will be assessed and compared to the individual or bulk gentamicin counterparts. The quartz crystal microbalance with dissipation monitoring (QCM-D) will be utilized to evaluate, in real time and under dynamic conditions, the interaction and sensitivity of the sessile *P. aeruginosa* cells with GeN_AC NSs. Finally, the NSs toxicity and the functional stability in protein-enriched conditions, mimicking the in vivo application scenario, will be evaluated.

## 2. Results and Discussion

### 2.1. Hybrid GeN_AC NSs Characterization

**Figure 1:**
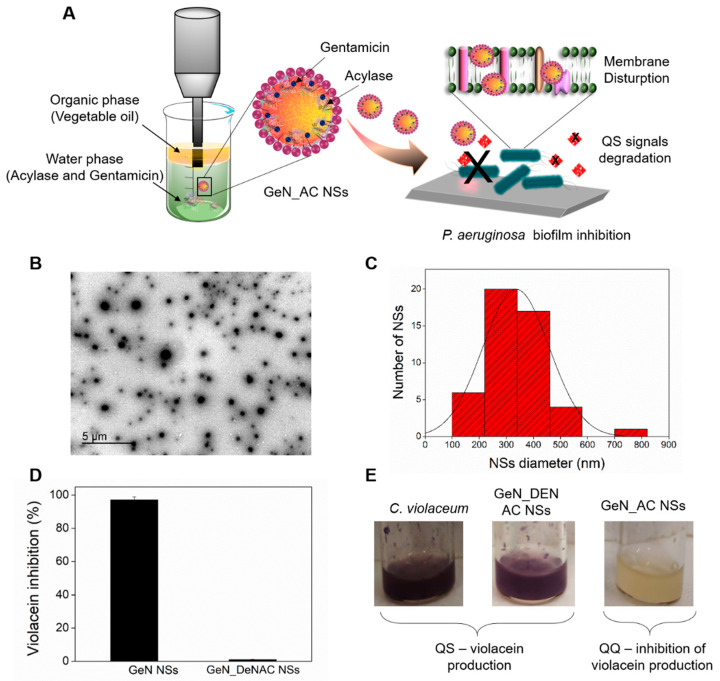
(**A**) Schematic representation of US-assisted generation of antibacterial gentamicin and QQ acylase NSs and their interaction with *P. aeruginosa* planktonic and sessile cells; “X” represents the degradation of QS signals and inhibition of biofilm formation by acylase (**B**) TEM image of GeN_AC NSs and (**C**) corresponding histogram of the size distribution based on the total count of 100 NSs using ImageJ software; (**D**) Quantification of violacein production by *C. violaceum* and (**E**) representative images of violacein production in the presence of GeN_DeNAC NSs and GeN_AC NSs.

One-pot ultrasonic nano-emulsification was used to generate oil-filled hybrid NSs in which the antibiotic and enzymes are located at the interface of the organic droplet (Figure 1A). Ultrasound-induced nano-emulsification is a simple, safe, environmentally friendly and energy-efficient method, extensively employed in our group for producing NSs of different actives, including antibiotics [17,18], polysaccharides [20] and proteins [21] with an enhanced efficacy compared to their bulk counterparts. The high-intensity ultrasonication of the oil–water interface generates acoustic cavitation, comprising the sudden formation (nucleation), growth (expansion) and implosive collapse of micron-sized bubbles forming stable nano-emulsions [22].

The sonochemically generated GeN_AC NSs, with an average particle size of 206 ± 1 nm and ζ-potential of −33.45 ± 0.92 mV, demonstrated high physical stability and did not aggregate or precipitate in solution for up to 6 months stored at 4 °C. The high colloidal stability of the hybrid NSs is explained with the use of a non-ionic surfactant, Tween 80, during the NSs formulation [23]. Nanoparticle tracking analysis (NTA) revealed the formation of homogeneous NSs with a low polydispersity, narrow size distribution profile and concentration of 2.2 × 10^13^ ± 0.04 NSs mL^−1^ (Figure S1, Supplementary Materials). The sphere-like shape and size of the GeN_AC NSs in the range between 60 and 140 nm were observed by transmission electron microscopy (TEM) (Figure 1B,C).

To assess the amount of the encapsulated gentamicin in the NSs, we further used high-performance liquid chromatography (HPLC) coupled to a UV-Vis detector. Prior to the HPLC–UV-Vis analysis, GeN and GeN_AC NSs were separated from the bulk solution by ultracentrifugation, cleaned from the oil phase and chemically derivatized with a chromophore (o-phthalaldehyde). The chromatographic analysis revealed 32% gentamicin transformation into NSs. A Lowry protein assay of the demulsified NSs confirmed 87% encapsulation efficiency of the acylase. The inclusion of acylase in the NSs was also demonstrated by the appearance of a green fluorescence signal in the NSs, prepared using previously fluorescein isothiocyanate (FITC)—labelled enzyme (Figure S2, Supplementary Materials).

Considering the fact that the sonication may negatively affect the acylase conformation, its catalytic activity was assessed. The development of a purple color in the ninhydrin colorimetric assay confirmed the degradation of the N-acetyl-L-methionine substrate to L-methionine by acylase and thus its ability to degrade the amide bonds in the QS-signaling molecules after nano-spherization. The NSs retained 98% of the acylase activity when compared to the protein in its bulk form (Figure S3, Supplementary Materials). To confirm the quorum quenching (QQ) potential of the nano-formulated acylase we also used *Chromobacterium violaceum* (*C. violaceum*) CV026, a mini-Tn5 mutant of the wild-type *C. violaceum* that produces a purple pigment, called violacein, in the presence of AHLs [12,16,24]. In this assay, GeN_AC NSs were applied at sub-inhibitory concentrations (0.0625 × 10^13^ NSs mL^−1^) that do not affect the *C. violaceum* viability (Figure S4, Supplementary Materials), but rather the cell density depended virulence factors production controlled by the QS process. As a result of the AHL degradation by acylase, the QS-regulated violacein secretion by *C. violaceum* was inhibited by 97 ± 0.003% (Figure 1D). On the contrary, the hybrid NSs prepared with non-active QQE (GeN_DeNAC NSs) did not reduce the purple pigment production, confirming the acylase-induced interruption of the QS process via degradation of AHL signals (Figure 1D,E).

### 2.2. Antibacterial Activity of GeN_AC NSs against P. aeruginosa

**Figure 2:**
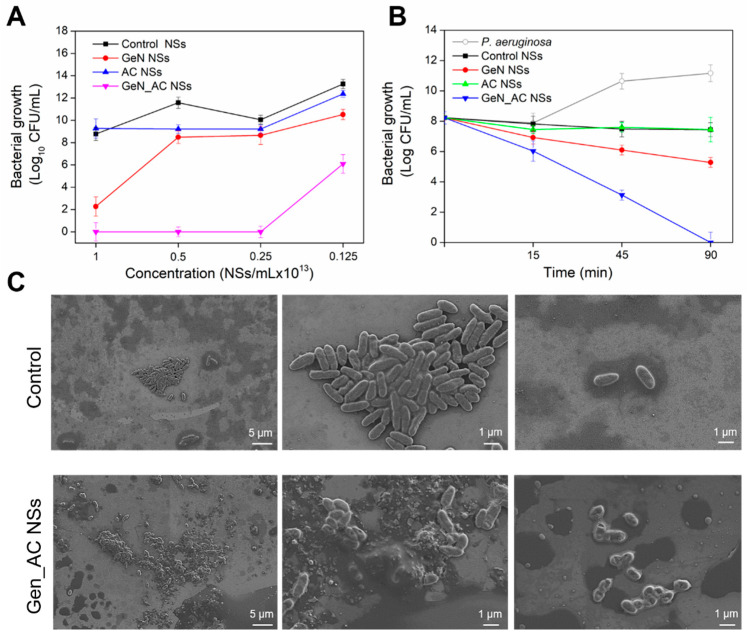
(**A**) *P. aeruginosa* growth after 24 h incubation with different concentrations of control, AC, GeN and GeN_AC NSs; (**B**) Time-kill kinetics of control, AC, GeN and GeN_AC NSs; (**C**) Scanning electron microscopy images of *P. aeruginosa* in absence and presence of GeN_AC NSs (0.25 × 10^13^ NSs mL^−1^) at different magnification.

The antibacterial efficacy of the hybrid GeN_AC NSs, and the individual AC NSs and GEN NSs, was studied against *P. aeruginosa*. Since acylase is a QQE, interfering only with the QS process in *P. aeruginosa* through AHL signals’ hydrolysis without affecting the bacterium viability [12], the developed AC NSs did not affect the live bacterial cells’ count. On the other side, the nano-formulated gentamicin, in a concentration 1 × 10^13^ NSs mL^−1^, reduced the *P. aeruginosa* growth by six logs. The nano-hybridization of the antibiotic with QQ acylase, however, resulted in enhanced bactericidal activity and complete bacterial eradication at even lower NSs concentration (0.25 × 10^13^ NSs mL^−1^), due to the synergistic mode of anti-*P. aeruginosa* action comprised of: (i) attenuation of the bacterial virulence via acylase-induced interruption of QS; and (ii) nano-form enhancement of the antibiotic bactericidal activity (Figure 2A). The nano-formulation of antimicrobials drastically changes their physical properties, transforming these actives into more efficient bactericides compared to their non-processed bulk solutions, due to the improved membrane-disturbing effect [17,18]. In this work, nano-sized transformation of gentamicin led to four-fold improvement of its bactericidal activity, whereas the boosting effect of the QQE yielded a 16-fold higher efficacy compared to the bulk gentamicin (Figure S5, Supplementary Materials).

The time-kill kinetics of the hybrid NSs (at their minimum bactericidal concentration (MBC) of 0.25 × 10^13^ NSs mL^−1^) and corresponding controls were performed to define the pace at which the actives exerted their bactericidal properties. Complete bacterial eradication was achieved within 90 min for GeN_AC NSs, whereas for the same period the stand-alone GeN NSs did not show a growth reduction (Figure 2B). To prove that the enhanced killing effect of the antibiotic was due to its nano-hybridization with anti-QS acylase, hybrid GeN_DeNAC NSs, containing gentamicin and non-QS active enzyme, were generated. These NSs were of 201 ± 0.21 nm size and −31 ± 0.15 mV ζ-potential, similar to the QQ active hybrid NSs. The GeN_DeNAC NSs demonstrated lower antibacterial activity than the GeN_AC NSs, while the bacterial growth reduction was similar to the GeN NSs (Figure S6, Supplementary Materials). These results confirmed the importance of QQE for potentiating the efficacy of the nano-formulated gentamicin against *P. aeruginosa*.

The interaction of the GeN_AC NSs with *P. aeruginosa* was also assessed using scanning electron microscopy (SEM) (Figure 2C). The SEM images demonstrated that the control *P. aeruginosa* cells were intact and did not present any morphological changes. They appeared rod-shaped measuring 0.2 to 0.4 μm in width and 1 to 1.5 μm in length. Clear differences between the control and the bacteria treated with the GeN_AC NSs were observed. Changes in the surface morphology of the *P. aeruginosa* cells associated with cellular death were detected, which is in agreement with the results from the counting of the live bacterial cells (Figure 2A).

Gentamicin is an aminoglycoside antibiotic that binds to the 30S ribosomal subunit of bacterial cells, inhibiting protein synthesis in Gram-negative *P. aeruginosa* [25]. However, the SEM micrographs also revealed cell wall damages with pore formation, which suggested that the GeN_AC NSs possess additional mechanisms of action, e.g., disruption of the bacterial cell membrane and therefore a lower probability of evoking resistance [26]. Previous works in our group have demonstrated that the nano-formulation endows the antibiotics, penicillin and vancomycin, with improved bactericidal activity at low amounts, explained by the improved physical interaction with the Gram-negative bacterial cell wall and easier access to the target site [17,18].

### 2.3. GeN_AC NSs Interaction with Biomimetic Bacterial Membranes

**Figure 3:**
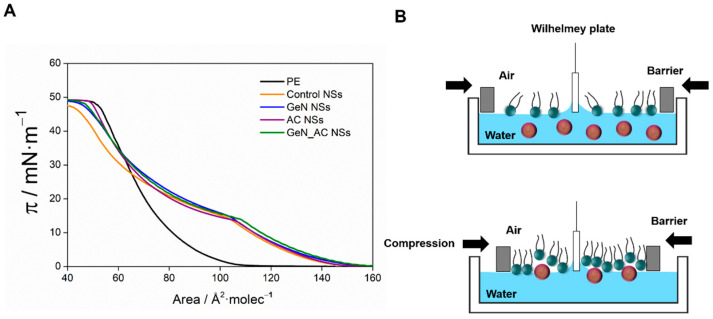
(**A**) Surface pressure-area isotherms of a PE monolayer (the area per molecule in X axis refers to *E. coli* PE) in water with control, AC, GeN and GeN_AC NSs; (**B**) Schematic representation of the NSs interaction with model bacterial membrane.

The mechanism of action of the novel GeN_AC NSs was studied by surface pressure-area isotherms of Langmuir films, in which phosphatidylethanolamine (PE) was used to prepare a Gram-negative bacterial membrane model [18,19]. The π–A isotherm for the PE, with only water in the subphase, showed a lift-off area of around 100 Å^2^ molecule^−1^, whereas the addition of the NSs to the subphase resulted in an increase in the molecular area to 140 Å^2^ molecule^−1^ (Figure 3). The expulsion of the control NSs from the PE monolayer induced a lower tilting of the isotherm at high surface pressure when compared with the neat PE, which indicates the presence of NSs in the monolayer even at a surface pressure close to the physiological membrane. Similar behavior has been previously observed for exogenous molecules inserted in the monolayers of galactolipids mimicking the thylakoid membrane [27,28]. Both the GeN and GeN_AC NSs caused an increase in the area per molecule at a low surface pressure compared with the isotherm of the control NSs, which is explained by the interaction between the gentamicin amino groups and PE. The partial expulsion of the gentamicin-containing NSs from the PE monolayer yielded an isotherm close in shape to the control NSs one, indicating a similar disturbing effect assigned to the non-ionic surfactant involved in the NSs stabilization. Therefore, the stronger antimicrobial effect of the hybrid NSs is likely due to the enhanced permeability and nano-form induced damage of the Gram-negative bacterial membrane, coupled with the gentamicin capability to inhibit protein synthesis.

### 2.4. Antibiofilm Activity of GeN_AC NSs

As the fact that the GeN_AC NSs were able to disturb the biomimetic membrane and kill the planktonic *P. aeruginosa* cells was proved, we then evaluated their potential to prevent biofilm formation. *P. aeruginosa* produces robust biofilm structures on a variety of surfaces, including medical devices (e.g., urinary catheters, implants, contact lenses, etc.) and host tissues and organs (e.g., lungs, urinary tract, respiratory pathway). The bacterium enclosure in an autogenic extracellular polymeric matrix protects the cells from antibiotics, impedes phagocytosis and frequently leads to long-term persistence. It is of key importance to inhibit the *P. aeruginosa* growth at an early stage, i.e., before the biofilm development, in order to enhance the susceptibility of this pathogen towards existing antimicrobial treatments.

**Figure 4:**
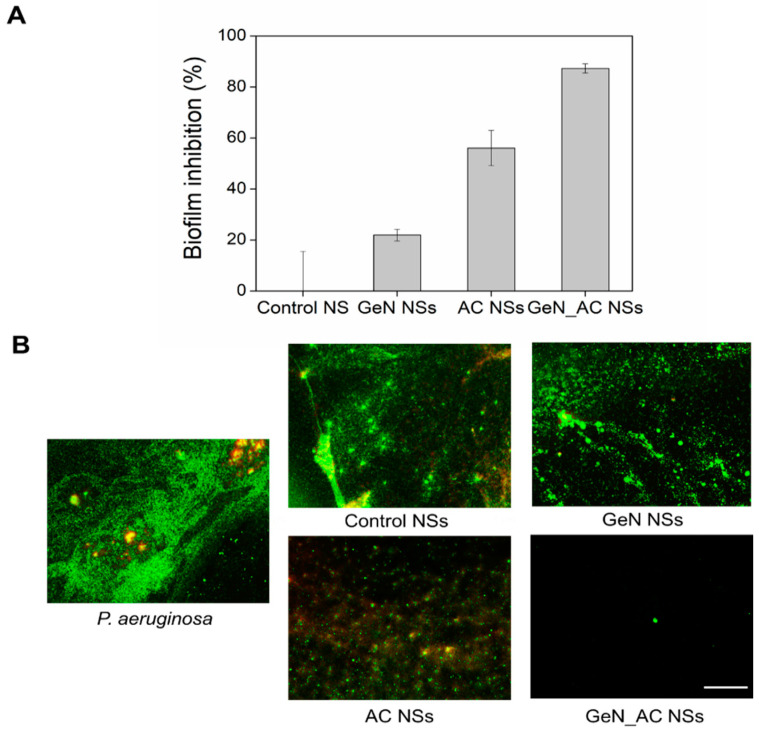
Inhibition of *P. aeruginosa* biofilms by control, AC, GeN and GeN_AC NSs (0.125 × 10^13^ NSs mL^−1^). (**A**) Total biomass determination with crystal violet; (**B**) Fluorescence microscopy images of live (green) and dead (red) bacteria in the biofilms. The green and red fluorescence images are overlaid. Scale bar corresponds to 100 μm.

The developed nano-hybrids of acylase and gentamicin inhibited by up to 95% the total biomass establishment of *P. aeruginosa* at a concentration of 0.125 × 10^13^ NSs mL^−1^. In contrast, the stand-alone GeN and AC NSs reduced the biofilm mass by only 20% and 60%, respectively. We assume that the acylase in the hybrid NSs breaks down the 3-oxo-C12-HSL and C4-HSL signals, making the *P. aeruginosa* less virulent and unable to establish well-defined surface-attached biofilms, thus increasing the antibacterial effect of the nano-formulated gentamicin at a lower dosage (Figure 4A).

Microscopic images of the live and dead bacteria corroborated the aforementioned results (Figure 4B). Complete biofilm inhibition was achieved with the hybrid GeN_AC NSs, whereas in the case of the non-treated *P. aeruginosa*, a well-established biofilm structure embedded with green (live) and red (dead) cells was observed. At these concentrations, the stand-alone GeN NSs did not reduce the total biofilm mass, whereas the AC NSs showed a decrease in the *P. aeruginosa* biofilm formation, as previously observed in the crystal violet test (Figure 4A). Importantly, the control without the actives neither affected the total *P. aeruginosa* biomass formation, nor the viability of the biofilm cells. The red cells detected in the non-treated and treated biofilms may also result from the reaction of the matrix extracellular DNA, with propidium iodide used to assess the biofilm cells’ viability. These results proved that the acylase and antibiotics in the nano-hybrids impede the biofilm establishment simultaneously, interfering with the extracellular signaling in the bacterial community-acquired resistance and bacteria viability.

### 2.5. Real-Time Monitoring of the GeN_AC NSs Interaction with Sessile P. aeruginosa Cells

Taking into account the potential of the nano-formulated bactericides to interact with the bacterial biofilms and cells, leading to cellular lysis and death, we monitored in real time the effect of our GeN_AC NSs on the *P. aeruginosa* surface-attached cells under dynamic conditions, using QCM-D. This technique has been employed in our group to investigate the interaction of antibacterials with bacterial and mammalian mimetic membranes, as well as to monitor different stages of bacterial growth and biofilm formation including cells’ adherence, biofilm differentiation and eradication [29,30,31].

**Figure 5:**
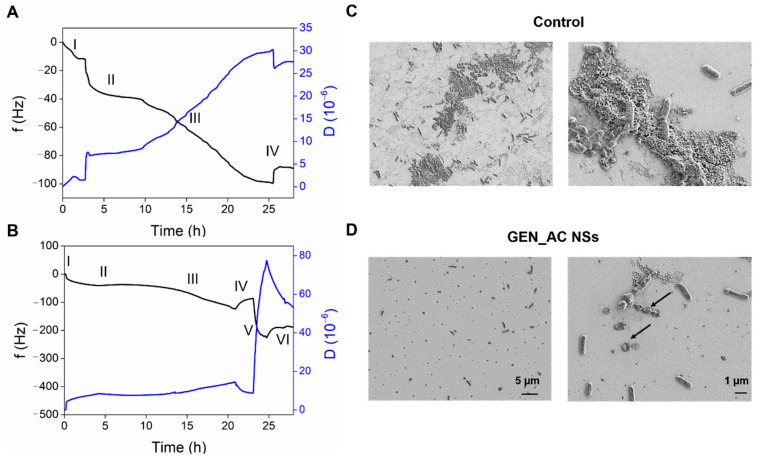
Interaction of GeN_AC NSs with *P. aeruginosa* assessed by QCM-D. (**A**,**B**) The shift in the frequency (Δf_5_) and dissipation (ΔD_5_) are represented with black and blue lines, respectively. The numbers indicate: I—the baseline establishment, II—QCM inoculation and III—growth of *P. aeruginosa* sessile cells, IV—baseline after removal of the loosely attached cells, V—insertion and circulation of GeN_AC NSs and VI—washing with PBS. SEM images of the QCM-D gold disks non-treated (**C**) and treated with GeN_AC NSs (**D**) at different magnification.

At first, the *P. aeruginosa* was allowed to settle onto the QCM sensor and the shifts in the frequency and dissipation of the seventh overtone during the GeN_AC NSs circulation were recorded (Figure 5). The QCM crystal inoculation with the bacterium at O.D.=0.1 led to a sudden decrease in the frequency (≈40 Hz) (Figure 5A,B, zone II) from the baseline established with the 100 mM phosphate buffered saline, pH 7.4 (PBS) (Figure 5A,B, zone I). This drastic alteration was related to the fast “bulk shift” caused by the change of the medium, and the rapid adherence of the tryptic soy broth (TSB) constituents and the *P. aeruginosa* cells onto the disk [29,31]. After 3 h of *P. aeruginosa* seeding (Figure 5A,B, zone II), the bacteria were left overnight under a constant flow of fresh TSB in order to obtain well-defined colonies of surface-attached cells for further interaction with the GeN_AC NSs (Figure 5A,B, zone III). Following a PBS rinse of the loosely bound cells (Figure 5A,B, zone IV), a slight frequency increase/dissipation decrease and the establishment of steady-state signals were obtained (Figure 5A,B, zone V). Then, the injection of the GeN_AC NSs into the QCM chamber caused a significant decrease in the frequency (250 Hz) and increase in the dissipation, not observed for the control treatment of the *P. aeruginosa* with PBS without NSs (Figure 5B, zone VI). The changes in the frequency were assigned to NSs deposition and interaction with the bacterial cells grown onto the gold crystal, while the dissipation shifts were ascribed to the increased water content, the rearrangement of the surface-attached bacterial cells and changes in their morphology.

To better understand the effect of the GeN_AC NSs on the sessile *P. aeruginosa* cells, the treated and non-treated QCM disks were subjected to SEM analysis. The morphological changes induced by the GeN_AC NSs on the *P. aeruginosa* were in agreement with the detected dissipation changes confirming the bactericidal effect of the NSs. A significant reduction in the total biomass, when compared to the untreated gold disk, was also observed (Figure 5C,D). Although the GeN_AC NSs do not possess biofilm-degrading activity, we assume that the GeN_AC NSs attach to and migrate within the overnight-grown micro-colonies of bacteria and extracellular components to better interact with the cells. The bacterial viability reduced by the gentamicin together with the NSs-induced physical perturbation of the *P. aeruginosa* surface-adhered clusters, ultimately resulted in the weakened attachment and easier flushing of the bacterial cells and extracellular components by the flow.

### 2.6. Biocompatibility of GeN_AC NSs

**Figure 6:**
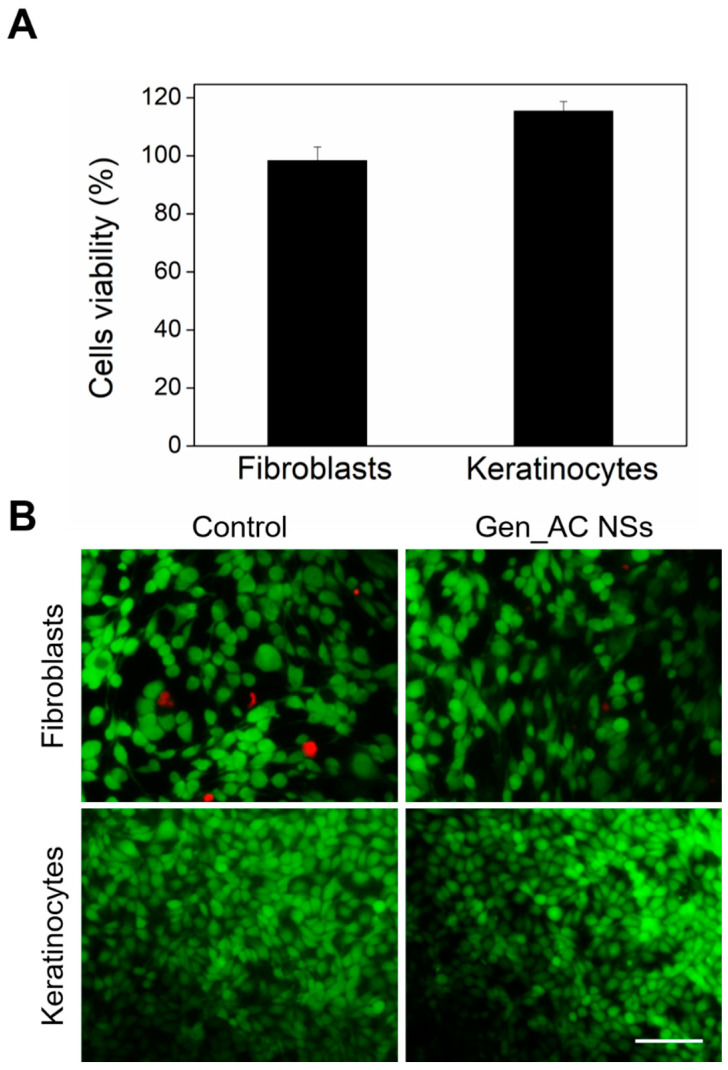
(**A**) Relative viability of HaCaT and BJ-5ta cell lines after 24 h exposure to bactericidal concentrations (0.25 × 10^13^ NSs/mL) of GeN_AC NSs determined by AlamarBlue™ assay; (**B**) Fluorescent microscopy images of HaCaT and BJ-5ta cell lines after 24 h of exposure to GeN_AC NSs. The green (live cells) and red (dead cells) fluorescence images are overlaid. Scale bar corresponds to 100 μm.

Since the materials at a nanometer scale are highly active, it is important to ensure that they do not cause detrimental effects to human cells. The potential toxicity of the GeN_AC NSs was evaluated towards fibroblasts and keratinocytes human cell lines (Figure 6). The GeN_AC NSs at 0.25 × 10^13^ NSs mL^−1^ were placed in contact with the cells for 24 h and then the human cells’ viability was assessed. AlamarBlue™ and Live/Dead™ viability assays, showing more than 95% of both the keratinocytes and the fibroblasts survival (Figure 6A), most of them stained in green (Figure 6B), respectively, confirmed that the GeN_AC NSs were safe for the human cells at bactericidal concentrations.

### 2.7. Influence of Protein Corona Effect on the Functional Properties of GeN_AC NSs

The stability and functionality of the nano-formulated antibacterial actives, in conditions mimicking the non-specific protein adsorption on their surface during circulation in vivo, the so-called “protein corona”, are critical parameters to be considered in biomedical applications. The protein corona phenomenon affects the nano-agents activity and toxicity, and is generally associated with the deposition of serum proteins onto the nano-entities when they are in contact with biological fluids [32,33].

**Figure 7:**
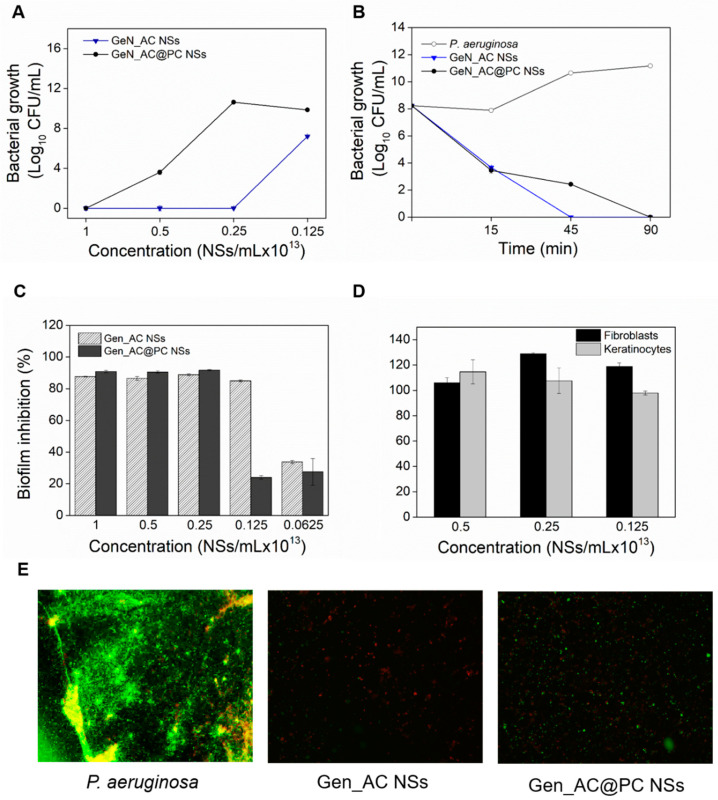
Protein corona effect on GeN_AC NSs functionalities. (**A**) *P. aeruginosa* growth after 24 h incubation with different concentrations of protein corona and non-protein corona GeN_AC NSs; (**B**) Time–kill curves of GeN_AC NSs before and after incubation in protein-rich conditions; Inhibition of *P. aeruginosa* biofilms by GeN_AC NSs and GeN_AC@PC NSs assessed with (**C**) crystal violet and (**E**) live/dead cells viability kit. The green (live cells) and red (dead cells) fluorescence images are overlaid. Scale bar corresponds to 100 μm; (**D**) Relative viability of HaCaT and BJ-5ta cell lines after 24 h of exposure to varying concentrations (1, 0.5, and 0.25 × 10^13^ NSs mL^−1^) of GeN_AC@PC NSs.

Herein, the exposure of the GeN_AC NSs to serum-enriched conditions resulted in a negligible decrease in their surface charge (from −33.45 ± 0.92 mV to −24.85 ± 0.64 mV) and an increase in their hydrodynamic diameter (from 206 ± 1 nm to 260 ± 0.5 nm). The serum proteins are mostly negatively charged at a physiological pH and, therefore, an increased repulsion towards the GeN_AC NSs might be the reason for the attenuated protein corona effect [33]. Nevertheless, the changes in the NSs size indicated other types of non-electrostatically driven interactions, such as hydrogen and hydrophobic bonding with serum proteins [33]. The non-specific protein adsorption on the GeN_AC NSs caused slight changes in their antibacterial and antibiofilm activities. The protein corona NSs (denoted as GeN_AC@PC NSs) demonstrated a two-fold decrease in the antibacterial activity when compared with the non-protein corona GeN_AC NSs (Figure 7A). Additionally, time–kill curves of GeN_AC@PC NSs and GeN_AC NSs at their bactericidal concentrations revealed deviations in the NSs pharmacodynamics by the serum proteins. Complete bacterial eradication (eight log reduction) was achieved 90 min after the incubation of GeN_AC@PC NSs with the *P. aeruginosa*, whereas only 45 min were needed for the GeN_AC NSs to achieve the same effect (Figure 7B). The bacterial killing rates are very dependent on the type of bactericidal active and its concentration. We consider that the slower gentamicin release from the GeN_AC NSs decorated with serum proteins might be the reason for their lower killing rate [34].

A decrease in the antibiofilm efficacy of the GeN_AC@PC NSs was also detected (Figure 7C–E). A complete inhibition of the *P. aeruginosa* biofilm, in terms of total biomass and viable biofilm cells reduction, was obtained in two-fold higher concentrations than the non-protein corona GeN_AC NSs (Figure 7C,E). The non-specific protein attachment to the enzyme protein may also impact on the catalytic activity of acylase, and consequently reduce the NSs anti-infective and antibiofilm potential.

Despite the observed efficacy decrease in the hybrid GeN_AC NSs in conditions mimicking their circulation in the bloodstream in vivo, these were still highly effective against the *P. aeruginosa* planktonic and sessile cells at safe to human cells dosages (Figure 7D). Therefore, the nano-hybridization of antibiotics with QQE is an innovative approach for boosting the activity of bactericides with great potential for managing *P. aeruginosa* urinary tract, lung and skin infections.

## 3. Materials and Methods

### 3.1. Materials, Reagents and Bacteria

The acylase from *Aspergillus melleus* (specific activity of 0.25 U mg^−1^), Luria Bertani broth (LB)—for acylase QQ activity evaluation on *C. violaceum*, Mueller Hinton broth (MHB)—for determining minimal inhibitory/bactericidal concentrations, TSB—for biofilm inhibition tests and cetrimide selective agar for the culturing and enumeration of *P. aeruginosa* were purchased from Sigma-Aldrich, Madrid, Spain. The L-α-phosphatidylethanolamine (PE) was purchased from Avanti Polar Lipids, Inc. (Alabaster, AL, USA). The Live/Dead^®^ BacLight kit™ (Molecular probes L7012) and AlamarBlue™ reagent—for bacterial and human cells viability tests, respectively, were obtained from Invitrogen, Life Technologies Corporation (Sant Cugat del Vallès, Spain). The *P. aeruginosa* ATCC 10145, human foreskin fibroblasts (BJ-5ta cell line) and keratinocytes (HaCaT cell line) were obtained from the American Type Culture Collection (ATCC LGC Standards, Barcelona Spain). The Live/Dead^®^ kit for mammalian cells viability was obtained from Thermo Fischer Scientific (Sant Cugat del Vallès, Spain). All of the other reagents were purchased from Sigma-Aldrich (Spain), unless otherwise specified. Ultrapure MilliQ water with a resistivity of 18.2 MΩ cm was used in all of the experiments.

### 3.2. Ultrasound-Assisted Preparation of GeN_AC NSs

Hybrid GeN_AC NSs and stand-alone AC and GeN NSs were produced in an one-step ultrasound emulsification process, as previously described [18]. Briefly, 1 mg mL^−1^ of acylase and/or 50 µg mL^−1^ of gentamicin were dissolved in MilliQ water containing 0.1% (*v*/*v*) Tween 80. Then, 30 mL of the enzyme/antibiotic solution (the aqueous phase) and 12 mL of commercial sunflower oil (the organic phase) were placed into a thermostatic sonicator cell (8 °C ± 1 °C) and the bottom of the Ti horn probe was positioned at the aqueous–organic interface. The NSs were produced for 3 min using a high-intensity Vibra-Cell VCX 750 ultrasonic processor (Sonics & Materials, Inc., Newtown, CT, USA) at 20 kHz and 35 % amplitude. The resulting NSs were subjected to centrifugation at 1500 rpm for 15 min to remove the non-encapsulated organic phase. An additional centrifugation step at 18,500× *g* for 40 min was performed to separate the bulk acylase and gentamicin from the developed NSs. The control NSs of only 0.1% (*v*/*v*) Tween and vegetable oil, without the active agents, were also produced. 

### 3.3. GeN_AC NSs Characterization

The NSs concentration was determined using NTA. NTA was performed in flow mode with a NanoSight NS 300 (Malvern Instruments Inc., Malvern, UK) equipped with a sCMOS camera. All of the experiments were carried out in quintuplicate. The size and Zeta potential measurements were carried out using a Zetasizer Nano ZS (Malvern Instruments Inc., Malvern, UK). The reported average particles’ size represents the mean values ± standard deviations (SD) of five measurements per sample. The size and the morphology of the NSs were also examined by TEM (JEOL JEM-2100 LaB6), operating at an accelerating voltage of 200 kV using SiO_2_ grid. The sample was prepared by dropping the NSs solution on the grids.

The amount of gentamicin transformed into the Gen NSs and GeN_AC NSs was quantified by HPLC. A 1200 HPLC system (Agilent Technologies, Madrid, Spain), consisting of a degasser, a quaternary pump and an automatic sample injector, coupled with a diode array detector for monitoring and recording the absorption spectra and chromatograms of the samples, was used for the gentamicin determinations. Agilent ChemStation software was used for data acquisition and processing. The chromatographic separation was performed on a Zorbax C-18 reserved-phase column (150 × 4.6 mm, 5 µm particle size) at 1 mL min^−1^ flow rate and isocratic conditions. The mobile phase was prepared by dissolving sodium 1-heptanesulfonate to 0.5% *w*/*v* in the mobile phase containing methanol, water and glacial acetic acid (70:25:5 *v*/*v*). All of the samples and controls were derivatized with o-phthaldialdehyde prior to chromatographic analysis. A total of 400 µL of each tested solution was transferred to a glass vial and mixed with 440 µL of isopropanol and 160 µL of derivatization mixture. The latter was prepared by dissolving 1 g of o-phthaldialdehyde in 5 mL methanol, adding 95 mL of 0.4 M boric acid in 8 M potassium hydroxide, pH 10.4 and 2 mL of thioglycolic acid. The amount of gentamicin encapsulated in the NSs was estimated, following the standard addition method. First, the NSs were separated (1 mL) from the suspension by ultracentrifugation (18,000 rpm for 45 min at 4 °C). Then, 2.5, 5 and 7.5 μL of 10 mg/mL of the gentamicin solution were added to each sample. All of the mixtures were vortexed and freeze-dried. After the oil phase removal, they were resuspended in 400 μL of MilliQ water and derivatized for their analysis, through the HPLC–UV-Vis system. The calibration curves were obtained by plotting the peak area of gentamicin absorption signals at 340 nm against its added standard gentamicin concentration. The AC NSs and control NSs were also analyzed, and any interfering peaks were found in the chromatographic separation at the gentamicin retention time.

### 3.4. Catalytic Activity of Acylase

After the NSs formation, the acylase activity was determined using a standard colorimetric method, as described before [16]. Briefly, 200 µL of the GeN_AC NSs were mixed with 200 µL of 100 mM Tricine buffer pH 8, 200 μL of 0.5 mM cobalt chloride solution and 200 μL of 0.1 mM N-acetyl-methionine (NAMET) and incubated at 37 °C for 30 min. The amount of the methionine produced due to the NAMET degradation by active acylase was assessed with ninhydrin assay. In this assay, 200 μL of the sample were mixed with 400 μL of 2% (*w*/*v*) of the ninhydrin solution and 20 μL of 1.6% (*w*/*v*) of the stannous chloride solution. The samples were incubated for 20 min at 100 °C and cooled down before measuring their absorbance at 570 nm. The GeN NSs and control NSs were used as the controls.

The ability of the nano-formulated acylase to degrade the AHL signals was further evaluated using *C. violaceum* CECT 5999, as previously described [12,16]. The *C. violaceum* was grown overnight in LB medium, supplemented with 25 μg mL^−1^ of kanamycin at 26 °C with agitation. For the QQ activity tests, the bacterium was diluted to optical density at 660 nm of 0.004 in fresh LB supplemented with 25 μg mL^−1^ of kanamycin and 5 μM QS signal (N-hexanoyl-L-homoserine lactone). Then, 1 mL of the inoculum was mixed with 1 mL of the GeN_AC NSs, at their non-bactericidal concentrations, or the control GeN_DeNAC NSs prepared with non-active acylase. The samples were incubated at 26 °C for 14–16 h with shaking. The *C. violaceum,* incubated only with LB, was employed as a control (violacein production). Afterwards, 800 μL of each sample were incubated with 800 μL of 10% (*w*/*v*) sodium dodecyl sulfate. The samples were vortexed for 5 s and mixed with 1 mL 1-butanol to extract the produced violacein. The samples were then centrifuged (14,000 rpm for 10 min), and the organic phase with violacein was measured at 584 nm.

### 3.5. Minimum Bactericidal Concentration Determination

The minimum bactericidal concentrations (MBC) of the NSs was determined by the broth microdilution method [35]. Briefly, 50 μL of the *P. aeruginosa,* diluted to an O.D. = 0.01 at 600 nm (~10^5^−10^6^ colony forming units (CFU)/mL) in MHB, were mixed with 50 μL of the NSs at different concentrations, ranging from 1 to 0.125 × 10^13^ NSs mL^−1^. After 24 h of incubation, the suspensions were serially diluted in sterile PBS and sub-cultured onto cetrimide agar plates to determine the number of survived bacteria after the treatment. All of the antibacterial data represent mean values ± SD (*n* = 3).

### 3.6. Time-Kill Kinetics

A time–kill kinetics assay was further performed to evaluate the minimal time needed for the GeN_AC NSs to eliminate all of the bacterial cells. The overnight-grown *P. aeruginosa* culture was diluted in the PBS to an O.D. = 0.01 at λ = 600 nm (~10^5^−10^6^ CFU mL^−1^). Then, 250 μL of the culture was mixed with 250 μL of the samples, ~0.25 × 10^13^ NSs mL^−1^ (concentration, corresponding to MBC), and incubated at 37 °C with 230 rpm agitation. At different time points, 10 μL of the samples were diluted in the sterile PBS and plated on cetrimide agar to enumerate the survived bacteria, using the drop plate method. After 24 h incubation at 37 °C, the grown colonies were counted. The bacteria in the PBS were used as a negative control (no bactericidal activity).

### 3.7. Interaction with Biomimetic Membranes

The interaction of the obtained NSs with the bacterial cell membrane model was assessed, using Langmuir films. Surface pressure—Area (π–A) isotherms were performed in a Langmuir trough, equipped with two mobile barriers (KSV NIMA, model KN2002, Espoo, Finland) with a total area of 273 cm^2^ mounted on an antivibration table and housed in an insulation box at 23 ± 1 °C. The Langmuir trough was cleaned with CHCl_3_ and water. After the subphase addition, the surface was further cleaned by suctioning.

GeN, AC, GeN_AC and control NSs were diluted in a 100 mM Tricine buffer, pH 8, until a final concentration of 0.25 × 10^9^ NSs mL^−1^ and used as subphase for the evaluation of their interaction with the PE monolayers. After pouring the subphase into the through 35 µL of 0.5 mg mL^−1^ of PE solution prepared in CHCl_3_ were spread dropwise. Ten minutes after the evaporation of the CHCl_3_, the barriers were compressed at 25 cm^2^ min^−1^. The π–A isotherms were performed in triplicate.

### 3.8. Antibiofilm Activity of GeN_AC NSs

#### 3.8.1. Crystal Violet Assay for Total Biofilm Mass Quantification

A total of 50 μL of the NSs and 50 μL of the *P. aeruginosa* (O.D._600_ = 0.01) in TSB were mixed in a sterile 96-well microplate and then placed at 37 °C for 24 h to form the biofilm. After that, the loosely adherent bacteria were washed three times with 200 μL distilled H_2_O, and the biofilm was fixed at 60 °C for 60 min. The total biomass developed on the surface was quantified by crystal violet assay, as described previously [19]. Briefly, the biofilm was stained for 5−10 min with 200 μL of 0.1% (*w*/*v*) crystal violet solution, washed three times with distilled H_2_O, and dried at 60 °C. Next, 200 μL of 30% (*v*/*v*) acetic acid were added to dissolve the crystal violet. A total of 125 μL of each well were then transferred to a new 96-well microplate, and the absorbance was measured at λ = 595 nm. The amount of the dye is directly proportional to the total biomass on the surface, including bacteria cells and the extracellular matrix.

#### 3.8.2. LIVE/DEAD^®^ BacLight™ Bacterial Viability for Microscopic Evaluation of the Biofilms

The biofilm inhibition capacity of the NSs was analyzed using Live/Dead^®^ BacLight™ kit. The kit contains two nucleic acid stains: (i) Syto 9-green-fluorescent dye labeling all of the live bacteria in green; and (ii) propidium iodide-red-fluorescent dye for the visualization of all of the dead bacteria in red. The *P. aeruginosa* biofilm was allowed to grow for 24 h in the presence of the NSs, as described above. After washing with 200 µL of sterile PBS, the biofilms were stained with 10 µL with a mixture of both of the stains (1:1) for 15 min in the dark and observed using fluorescence microscopy at 480/500 nm for the Syto 9 and at 490/635 nm for the propidium iodide.

#### 3.8.3. Real Time Monitoring of GeN_AC NSs Effect on Sessile *P. aeruginosa* Cells

The antibacterial effect of the GeN_AC NSs on the sessile *P. aeruginosa* cells was followed in real time using QCM-D (E4 system, Q-Sense, Zurich, Sweden), equipped with Teflon tubing. Prior to the assay, the gold QCM disks (QSX 301, Q-Sense, Zurich, Sweden) were cleaned with acetone, ethanol and isopropanol for 10 min at 40 °C, using US bath and then dried with nitrogen. The disks were placed in the QCM-D flow chambers at 37 °C and left to thermally stabilize for 10 min, before starting the experiments. The tests were performed under dynamic conditions using a digital peristaltic pump operating in pushing mode for the solutions, that were injected into the sensor crystal chamber at 20 μL min^−1^. A stable baseline with sterile 100 mM PBS, pH 7.4, was acquired and after that the *P. aeruginosa* (O.D._600_ = 0.1) was seeded for 3 h. Fresh TSB was circulated for ≈15 h and sterile 100 mM PBS, pH 7.4, was flowed to remove the loosely deposited bacteria and establish a second baseline. The *P. aeruginosa* sessile cells were then treated with the GeN_AC NSs dissolved in 100 mM PBS, pH 7.4, for over ≈2 h. The control experiment with only PBS without the GeN_AC NSs was performed in parallel. The tests were carried out in triplicate. For simplicity, the normalized frequency (Δf/ν) and dissipation (ΔD) shifts as a function of time of one representative sample per experimental group (seventh harmonic) are shown.

The morphology and growth of the *P. aeruginosa* on the QCM crystal upon treatment with the GeN_AC NSs were assessed by scanning electron microscopy. At the end of the experiment, the QCM sensor disks were washed twice with 100 mM PBS, pH 7.4, and the cells were fixed with 4% (*v*/*v*) formaldehyde at 4 °C overnight. Then, the gold disks were washed with 100 mM PBS, pH 7.4 and sequentially treated with 25, 50, 75 and 96% EtOH for 10 min each. The treated and non-treated *P. aeruginosa* surface-attached cells were observed, using a field emission scanning electron microscope (SEM) at 1kV (Merlin Zeiss).

### 3.9. Biocompatibility Assessment

The human fibroblast (BJ-5ta cell line) and keratinocytes (HaCaT cell line) were used to determine the toxicity of the GeN_AC NSs, as previously described [31]. The GeN AC NSs (0.125 × 10^13^ NSs mL^−1^) were incubated for 24 h with the previously seeded cells. Then, the cells’ viability was assessed by using the AlamarBlue™ cell viability reagent and the Live/Dead™ viability/cytotoxicity kit.

### 3.10. Protein Corona Simulation

To assess the effect of the non-specific protein attachment on the GeN_AC NSs functionalities, 800 µL of the NSs (2 × 10^13^ NSs mL^−1^) were incubated with 200 µL fetal bovine serum at 37 °C for 10 min with shaking. The samples were then centrifuged at 18,000× *g* for 40 min at 4 °C to remove the loosely attached proteins. Thereafter, the NSs were resuspended in the 100 mM Tricine buffer, pH 8, and their size, antibacterial and antibiofilm activities, as well as toxicity, were assessed as described before and compared to the non-protein corona GeN_AC NSs.

## 4. Conclusions

In view of the growing concern about the rapid emergence and spread of drug-resistant pathogens, there is an urgent need to design novel and more effective antibacterials as an alternative to the existing drugs. Compounds that modulate bacterial QS have attracted significant interest in recent years as promising therapeutic agents for increasing the bacterial vulnerability to lower dosages of conventional antimicrobials. In this work, sonochemically-synthesized hybrid NSs of acylase and gentamicin demonstrated enhanced antibacterial and antibiofilm efficiency against *P. aeruginosa*, due to a synergistic mode of action comprising attenuation of the bacterial pro-pathological cell-to-cell communication and nanoform-induced damage of the bacterial membrane. Such mechanisms of bacterial eradication do not induce selective pressure and might hamper the acquisition of new resistant mechanisms. The novel nano-hybrids demonstrated improved physical interaction and disturbance of the bacterial membrane, leading to complete bacterial removal at a 16-fold lower dosage, when compared to the bulk gentamicin. Their potential to interrupt QS via the degradation of the AHLs signals in the extracellular space significantly reduced the *P. aeruginosa* virulence and formation of the resistant biofilm. Importantly, the nano-actives did not induce toxicity effects or changes in the morphology of human cells at their bactericidal concentration, validating their safety for in vivo application. The negligible effect of the serum proteins on the GeN_AC NSs antimicrobial and antibiofilm properties would not preclude their therapeutic efficacy when exposed to biological fluids in vivo. These hybrid NSs could be further exploited in the development of injectable antimicrobials, topical formulations for the treatment of skin infections, actives in hydrogel wound dressings, aerosols for the treatment of lung disorders or as coatings on medical devices and implants for infection prevention and spread. Altogether, we provided a promising nano(bio)-technological approach for enhancing the efficacy of clinically relevant antimicrobials against infections caused by Gram-negative *P. aeruginosa*.

## Data Availability

All data generated or analyzed during this study are included in this manuscript and its supplementary information files.

## Supplementary Materials

The following supporting information can be downloaded at: https://www.mdpi.com/article/10.3390/ijms23147632/s1.
