# Supplementary material for: Nano-Formulation Endows Quorum Quenching Enzyme-Antibiotic Hybrids with Improved Antibacterial and Antibiofilm Activities against Pseudomonas aeruginosa
Source: Int J Mol Sci. 2022 Jul 11;23(14):7632. doi: 10.3390/ijms23147632 (PMC9321661; doi:10.3390/ijms23147632)
Supplement: Supplementary file 1 [file ijms-23-07632-s001.zip › ijms-1802988-supplementary.pdf]

International Journal of *Molecular Sciences*

MDPI

Supplementary Materials

Nano-formulation endows quorum quenching enzyme-antibiotic hybrids with improved antibacterial and antibiofilm activities against *Pseudomonas aeruginosa*

Kristina Ivanova [1], Aleksandra Ivanova [1], Javier Hoyo[1], Silvia Pérez-Rafael[1] and Tzanko Tzanov[1,*]

1 Grup de Biotecnologia Molecular i Industrial, Department of Chemical Engineering, Universitat Politècnica de Catalunya, Rambla Sant Nebridi 22, 08222, Terrassa, Spain; kristina.ivanova@upc.edu (K.I.); aleksandra.asenova@upc.edu (A.I.); javier.hoyo@upc.edu (J.H.); silvia.perez.rafael@upc.edu (S.P.-R.)

* Correspondence: tzanko.tzanov@upc.edu;

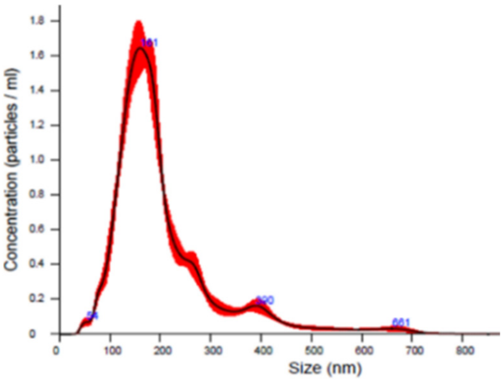

Figure S1. NTA analysis of GeN_AC NSs size distribution.

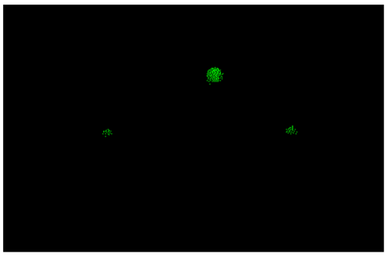

Figure S2. Florescence microscopy images of FITC-labelled acylase NSs.

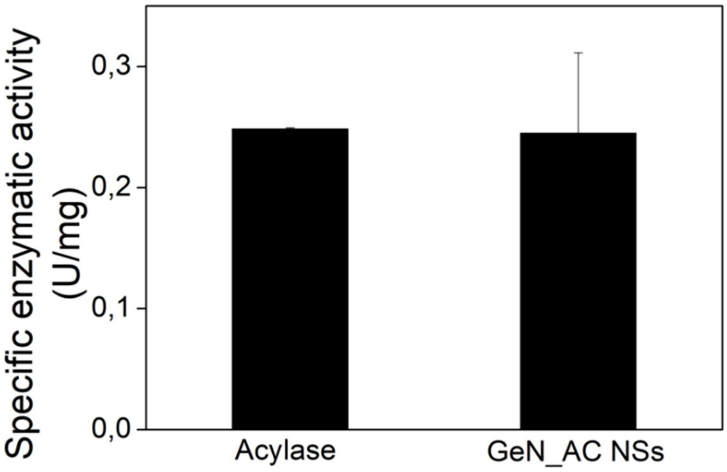

Figure S3. Acylase activity assessed by ninhydrin assay.

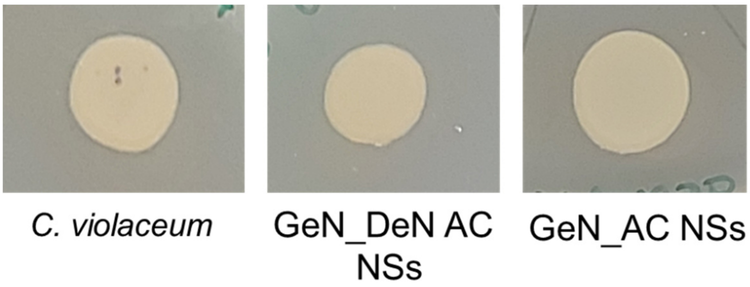

Figure S4. *C. violaceum* growth without and in presence of GeN_AC NSs and GeN_DeNAC NSs.

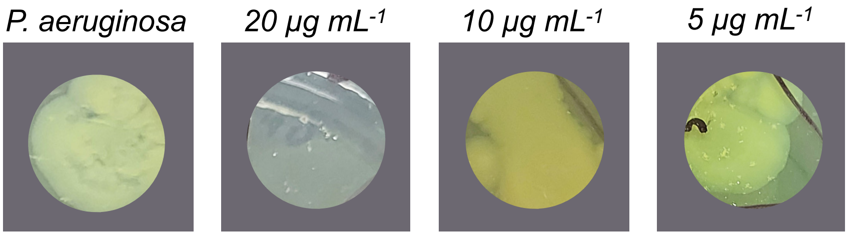

Figure S5. *P. aeruginosa* viability in presence of varying concentration of bulk gentamicin solution (5 – 20 μg mL^{-1}).

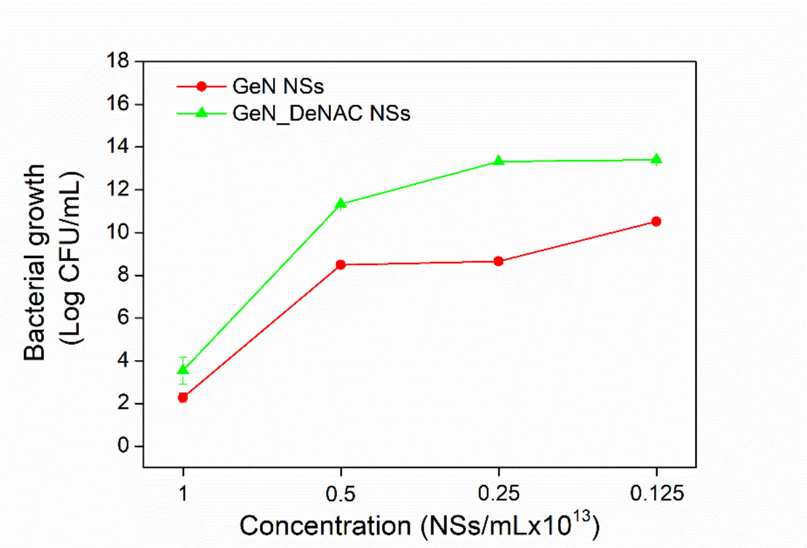

Figure S6. Antibacterial activity of GeN_AC NSs and GeN_DeNAC NSs against *P. aeruginosa*.
